# Effects of whole-body cryostimulation on spinal and shoulder range of motion in individuals with obesity

**DOI:** 10.3389/fresc.2025.1568280

**Published:** 2025-07-23

**Authors:** Serena Cerfoglio, Federica Verme, Jacopo Maria Fontana, Angelo Alito, Manuela Galli, Paolo Capodaglio, Veronica Cimolin

**Affiliations:** ^1^ Department of Electronics, Information and Bioengineering, Politecnico di Milano, Milan, Italy; ^2^ Laboratory of Biomechanics, Rehabilitation and Ergonomics, Istituto Auxologico Italiano, IRCCS, S. Giuseppe Hospital, Piancavallo, Italy; ^3^ Department of Biomedical, Dental Sciences and Morphological and Functional Images, University of Messina, Messina, Italy; ^4^ Department of Biomedical, Surgical and Dental Sciences, University of Milano, Milan, Italy

**Keywords:** whole-body cryostimulation, rehabilitation, obesity, range of motion, biomechanics

## Abstract

**Introduction:**

Flexibility and mobility are essential components of physical fitness, impacting joint function and musculoskeletal health. Individuals with obesity often exhibit restricted range of motion (ROM), exacerbated by muscle weakness, joint stiffness, and altered posture. Whole-body cryostimulation (WBC), involving exposure to low temperatures, has shown promise in alleviating inflammation and improving physical performance. This study evaluates the acute and short-term effects of WBC combined with rehabilitation on spinal and shoulder ROM in individuals with obesity.

**Methods:**

This non-randomized controlled trial included 42 adults with obesity undergoing a 4-week multidisciplinary rehabilitation program consisting of nutritional intervention, psychological support, physiotherapy, and physical activity. Participants were divided into two groups: a WBC group [WG, *n* = 21; 12 males (BMI = 38.77 kg/m^2^), 9 females (BMI = 38.45 kg/m^2^)] receiving 10 sessions (−110°C, 2 min/session) alongside rehabilitation, and a control group [CG, *n* = 21; 12 males (BMI = 43.37 kg/m^2^), 9 females (BMI = 41.86 kg/m^2^)] receiving rehabilitation alone. ROM for spine-related (i.e., anterior flexion, lateral bending, rotation) and shoulder-related (i.e., frontal rise, lateral rise, backward push) motor tasks was assessed at different time-points using a marker-based optoelectronic motion capture (MoCap) system. Repeated-measures (RM-ANOVA) analyzed changes within groups over time, *post hoc t*-tests identified significant effects, and mixed RM-ANOVA compared changes between groups.

**Results:**

Except for anterior flexion, WG showed significant improvements (*p* < 0.05) in ROM and task duration across all movements, with moderate to large effect sizes (0.20 ≤ *d* < 0.80). For instance, improvements were reported for ROM in shoulder flexion (acute-post: *p* = 0.045, *d* = 0.43) and extension (pre-post: *p* < 0.001, *d* = 0.51), as well as for spinal rotation (pre-post: *p* = 0.029, *d* = 0.42). Similarly, task duration reductions across all tasks, such as spinal rotation (pre-post: *p* = 0.040, *d* = 0.45) and lateral bending (pre-post: *p* < 0.025, *d* = 0.54). Conversely, CG showed no significant ROM changes.

**Discussion:**

WBC, when combined with rehabilitation, led to significant improvements in spinal and shoulder ROM. Acute and short-term benefits were observed in ROM and task duration, supporting WBC as a valuable addition to rehabilitation for individuals with obesity.

**Clinical Trial Registration:**

https://clinicaltrials.gov/study/NCT05443100, identifier NCT05443100.

## 1 Introduction

Flexibility is an intrinsic property of body tissues that determines the range of motion (ROM) achievable at a joint or group of joints and is widely regarded as a fundamental component of physical fitness and overall musculoskeletal health (1). It plays a critical role in facilitating efficient movement, preventing injury, and maintaining functional independence (2, 3). At the joint level, maximum ROM is influenced by a combination of structural and functional factors, including the mechanical properties of bones, tendons, ligaments, and joint capsules, as well as neuromuscular control (4). Among these, tendon compliance—the ability of tendons to stretch under load— is particularly important in allowing smooth and efficient joint movement (5).

Neuromuscular regulation also plays a pivotal role in flexibility. The stretch reflex, a protective neuromuscular mechanism, further influences flexibility by triggering muscle contraction in response to rapid elongation, thus preventing overstretching and potential injury (6). Furthermore, reduced neural activation decreases resistance to muscle elongation, facilitating greater ROM under a given load (7). Conversely, heightened muscle tension or neural activation restricts ROM by increasing resistance to elongation (8, 9). However, training methods like static stretching and proprioceptive neuromuscular facilitation can desensitize this reflex, allowing greater ROM without compromising stability (10).

While flexibility refers to the passive capacity to achieve ROM, mobility encompasses the active control of ROM and integrates muscle function, strength, stability, and coordination. Adequate flexibility supports mobility, but the latter requires additional elements to ensure efficient movement in sports and daily activities. Poor mobility can lead to compensatory movement patterns, increasing injury risk and impairing performance. Thus, optimizing both flexibility and mobility through targeted interventions enhances physical function and reduces injury risk, particularly in athletic contexts (11).

This interplay between structural and neuromuscular factors underscores the complexity of flexibility and how these factors may interact and differ among individuals. However, its determinants remain incompletely understood, with significant variability influenced by factors such as age (12), physical activity levels, specific training, and overall health status (13).

A reduced ROM is particularly relevant in the context of obesity, which is associated with various musculoskeletal disorders, including spinal impairments and osteoarthritis (14). Being overweight or having obesity can significantly reduce joint flexibility (15), contributing to restricted spinal mobility and increased stiffness, particularly in the dorsal region (16). Musculoskeletal disorders, especially in the shoulder (17), are common, potentially due to compensatory postural changes in the spine (e.g., increased stiffness) (18) and upper limbs (e.g., restricted ROM at the elbow) (19). Reduced flexibility in the thoracolumbar region may also lead to postural alterations, especially during prolonged standing, which increases mechanical stress on the hip joints (20). These postural changes, including hyperextension of the lumbar spine, increased anterior pelvic tilt while standing and a generally limited ROM due to reduced mobility in the pelvic and thoracic regions, are commonly observed in individuals with obesity (21). Abdominal circumference and gravitational forces may further influence lumbar lordosis and its mobility during forward flexion or lateral bending and altering muscle dynamics, particularly in erector spinae muscles (22). This may impair the ability of these muscles to counteract anterior shear forces, compromising spinal stability. Such postural changes can exacerbate spinal loading, increased torque and shear forces at the L5-S1 level, potentially contributing to disc degeneration (23). Other factors, such as neuromuscular activation deficits and muscle fatigue, may also contribute to reduced spinal stability during movement (24).

The application of thermal agents such as heat or cold is popular in clinical and rehabilitative settings and have been explored to improve ROM. Altering tissue temperature can have a range of therapeutic effects through changes in metabolism, nerve transmission, pain modulation, hemodynamics, and mechanical properties (25). Heat therapies, including thermotherapy, warm water immersion, and the application of hot packs, have been shown to enhance tissue elasticity, improve blood flow, and promote muscle relaxation, which collectively contribute to increased flexibility and range of motion (26–29). While the literature has indicated potential benefits of cryotherapy on ROM (28), the evidence remains inconsistent and highlights the need for further investigation.

Whole-body cryostimulation (WBC) is a physical/medical treatment where the entire body is exposed to extremely low temperatures (between −110°C and −140°C) for a short period of time, within dedicated cryochambers. Emerging evidence demonstrates that WBC elicits systemic physiological responses, including reductions in inflammation and oxidative stress (30, 31), pain (32–35), and stress/anxiety symptoms (36, 37). These effects position WBC as a promising adjunct therapy for various medical and sports medicine applications. The analgesic and anti-inflammatory benefits of cryostimulation are mediated by a sudden thermal gradient that activates cutaneous thermal receptors. This results in a reduction in skin temperature, a slowing of nerve impulse propagation in pain-transmitting fibers, and a modulation of pain signals through inhibitory pathways. In addition, repeated WBC sessions lead to a decrease in the production of pro-inflammatory mediators and oxidative stress markers. These physiological changes contribute to an increase in parasympathetic activity, concomitant with a reduction in sympathetic nerve activity, which is manifested by a decrease in fatigue, a reduction in muscle tension, an alleviation of delayed onset muscle soreness, and an improvement in mood and depressive symptoms (38). Initially employed for rheumatoid arthritis pain management (39), WBC's clinical applications have expanded to encompass orthopaedic, neurological, metabolic, autoimmune, psychiatric, and sleep disorders (30, 40–45).

Recent studies indicate that WBC modulates inflammatory responses, alleviating joint pain (46) and fatigue (47), which supports its potential as a valuable approach to managing obesity-related symptoms and improving physical function (44). This aligns with findings from studies demonstrating WBC's beneficial effects on mobility and pain reduction in patients with various musculoskeletal conditions (37, 48–51). Notably, when combined with exercise or physical therapy programs, repeated WBC has been shown to improve spinal mobility, muscle strength, endurance, and reduce low back pain and inflammation in patients with conditions like osteoarthritis, multiple sclerosis, and ankylosing spondylitis (37, 49–52). Furthermore, improvements in subjective measures of pain, mobility, and quality of life following WBC treatments have been reported in patients with various musculoskeletal conditions (i.e., knee and hip osteoarthritis, spinal pain syndromes) and chronic pain conditions like fibromyalgia and rheumatoid arthritis (35, 43, 53–56). Notably, De Nardi et al. (57) demonstrated significant improvements in sit-and-reach ROM after a single session of WBC.

Further research is warranted to fully elucidate WBC's impact on ROM in individuals with obesity. Obesity alters body movement biomechanics, hindering the joints' physiological ROM and increasing the risk of musculoskeletal overload. This dysfunction contributes to the high incidence of musculoskeletal disorders in this population, ultimately reducing their capacity to accomplish daily life and occupational activities (58, 59). By improving ROM and alleviating inflammation, WBC may offer a promising approach to mitigating these issues, but further studies are needed to confirm its long-term benefits and effectiveness in this context.

Previous studies evaluating WBC's effects on this population have primarily relied on subjective measures [e.g., Visual Analog Scales (VAS)] and functional tests [e.g., Timed Up and Go (TUG), Six-Minute Walk Test (6MWT)]. As for ROM assessment, manual goniometry has limited accuracy, which may prevent the consistent detection of clinically significant changes. To address these limitations and enable precise quantification of ROM changes, this study aims to objectively assess spinal and shoulder active ROM in individuals with obesity using a 3D marker-based optoelectronic motion capture (MoCap) system.

Marker-based motion capture (MoCap) systems are widely regarded as the gold standard for motion analysis and functional motor assessment, owing to their high levels of accuracy and reliability, with Intraclass Correlation Coefficient (ICC) values typically ranging from 0.80 to 0.99, depending on the measurement parameter and the protocol employed (60–62). These systems utilize reflective markers placed on specific anatomical landmarks, allowing for precise tacking of human movement through multiple synchronized cameras. Such methodologies are foundational in fields such as biomechanics and rehabilitation, where accurate movement quantification is crucial for assessment and intervention.

By leveraging these advanced technologies, the study aims to provide insights into how WBC may enhance mobility in individuals with obesity. The hypothesis is that WBC may help address mobility limitations, ultimately improving functional outcomes. By quantitatively tracking these changes within a rehabilitation program combining physiotherapy interventions with WBC, the study aims to contribute to a deeper understanding of how WBC impacts movement efficiency and overall musculoskeletal health in this population.

## 2 Materials and methods

### 2.1 Participants

This non-randomized controlled trial investigated the impact of WBC on active ROM in the spine and shoulder in individuals with obesity. A MoCap system was used to objectively measure ROM across a set of six motor tasks targeting the spine and shoulder at multiple time points.

The study was conducted between September 2023 and October 2024. A total of 42 participants were recruited among patients with obesity following a 4-week multidisciplinary rehabilitation program at San Giuseppe Hospital (IRCCS Istituto Auxologico Italiano, Piancavallo, Italy). The participants were divided into two cohorts. The first cohort (WG) consisted of 21 individuals with obesity undergoing the rehabilitation program complemented by a cycle WBC. The second cohort, serving as the control group (CG) included 21 individuals matched by age, sex, and BMI, who underwent only the rehabilitation program. Participants' characteristics are summarized in Table 1.

**Table 1:** Mean anthropometric and clinical features of participants.

Variable	WBC group (WG)	Control group (CG)
Participants (M/F)	21 (12/9)	21 (11/10)
Age (years)	50.48 (13.64)	52.19 (14.65)
Body mass (kg) (Baseline)	114.61 (23.77)	123.03 (25.00)
Weight loss during rehabilitation (kg)	−5.30 (2.21)	−6.65 (3.13)
Height (cm)	168.39 (21.61)	167.11 (11.34)
BMI (kg/m^2^)	39.40 (5.03)	42.58 (5.42)

Values are expressed as mean and standard deviation (SD), except for the number of participants, which is divided into males (M) and females (F) for each group.

Participants were selected based on the following inclusion criteria: age ≥18 years, BMI >30 kg/m^2^, absence of acute cardiovascular, respiratory, or infectious diseases, and absence of neurological or musculoskeletal conditions impairing the ability to understand vocal cues or motor functions. Participants with genetic obesity disorders (e.g., Prader-Willi Syndrome) were excluded. For eligibility to WBC treatment, additional criteria were considered, including the absence of unstable hypertension, no cold intolerance, no claustrophobia, no recent modifications to their usual pharmacological treatment, and body temperature ≤37.5°C.

The study protocol was approved by the Ethics Committee of the Istituto Auxologico Italiano (code 2021_05_18_14) and conducted in accordance with the ethical standards of the Institute and the 1964 Helsinki Declaration and its subsequent amendments. The sample in this study is part of a larger cohort initially gathered to examine broader patterns in the effects of WBC on individuals with metabolic or neurological conditions, fibromyalgia, or healthy patients with normal or excess weight (study registration: NCT05443100).

Written informed consent was obtained from all participants prior to enrollment.

### 2.2 Therapeutic intervention

The multidisciplinary rehabilitation program included tailored nutritional interventions, psychological support, physiotherapy sessions and supervised physical activity throughout the hospital stay. Participants were provided with a balanced hypocaloric diet structured into three main meals – breakfast, lunch, and dinner - formulated to provide 18%-20% protein, 27%–30% fat, 50%–55% carbohydrates, and 30 g of fibers derived primarily from fresh vegetables. Physical rehabilitation involved two daily 60-minutes sessions including individualized, progressive aerobic exercise training, activities to enhance postural control, and gradually intensifying strength training exercises, all supervised by qualified physiotherapists.

**Figure 1:**
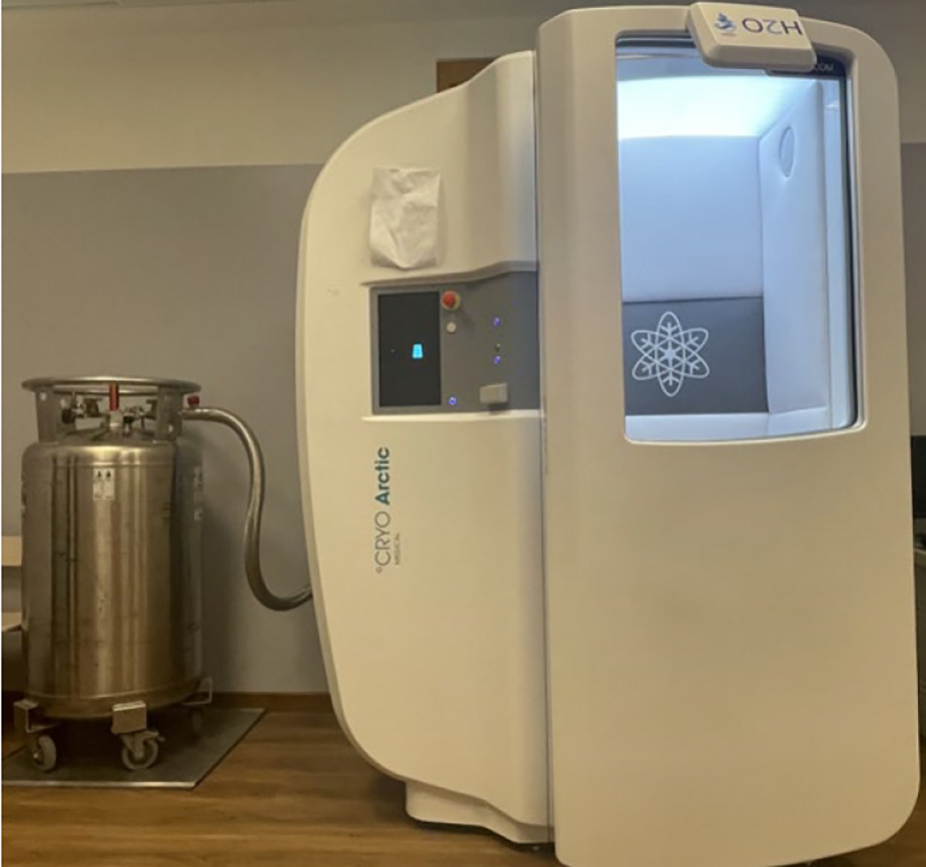
Visual representation of the cryochamber used in this study.

Participants in WG also underwent exposure to extremely cold, dry air (−110°C) in a cryochamber (Artic, CryoScience, Rome, Italy) where breathable air is present (Figure 1). Prior to treatment, patients wore minimal clothing, consisting of a surgical mask, ear band, gloves, a t-shirt, shorts, socks, and rubber clogs. Patients were also required to remove any glasses, contact lenses, and metallic jewelry, and ensure their skin was thoroughly dried to avoid skin burns. To familiarize patients with the procedure, a 1-minute trial session at −110°C was performed. Subsequent sessions (1st to 10th) were extended to 2 min at the same temperature. Throughout each session, continuous vocal and visual monitoring through the chamber's window was maintained to ensure patient comfort and safety. Each participant of WG completed a total of ten WBC sessions (i.e., 1 cycle), typically distributed over a two-week period. Skin temperature was assessed before and after the first and the last WBC sessions assessed using a high-precision infrared thermometer (Fluke 62 Max+, Fluke Corporation, Everett, WA, USA) with an accuracy of ±1°C or 1% of the measured value and a distance-to-spot ratio of 12:1, capable of detecting temperatures from −30°C to +650°C. Measurements were taken at four specific anatomical regions: nape of the neck (N), quadriceps (Q), popliteal cavity (PC), and calf (C). Skin temperature data were collected within one minute, both immediately before and after each WBC session.

### 2.3 Experimental set-up and study design for motor assessment

**Figure 2:**
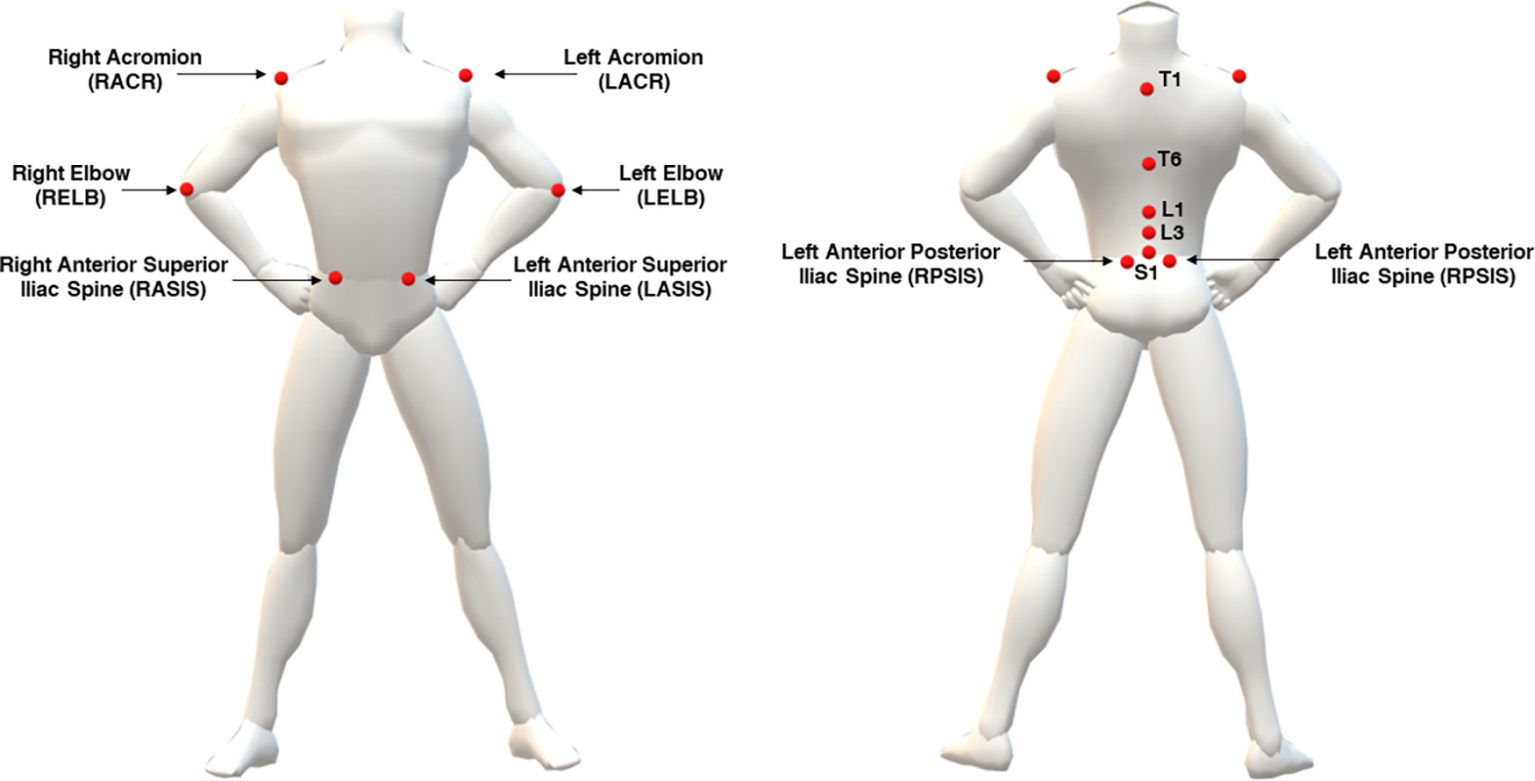
Placement of markers for tracking spine and shoulder motion.

All participants underwent instrumented motor assessment at the Motion Analysis Laboratory of San Giuseppe Hospital, equipped with a 6-camera, marker-based optoelectronic MoCap system (VICON, Oxford Metrics Ltd., Oxford, UK; sampling rate: 100 Hz). A set of 12 reflective makers (Ø = 15 mm) was manually placed on specific anatomical landmarks according to the model proposed by Menegoni et al. (63), with two additional markers placed on the right and left elbows to track upper limb motion (64). The full marker setup is as shown in Figure 2.

**Figure 3:**
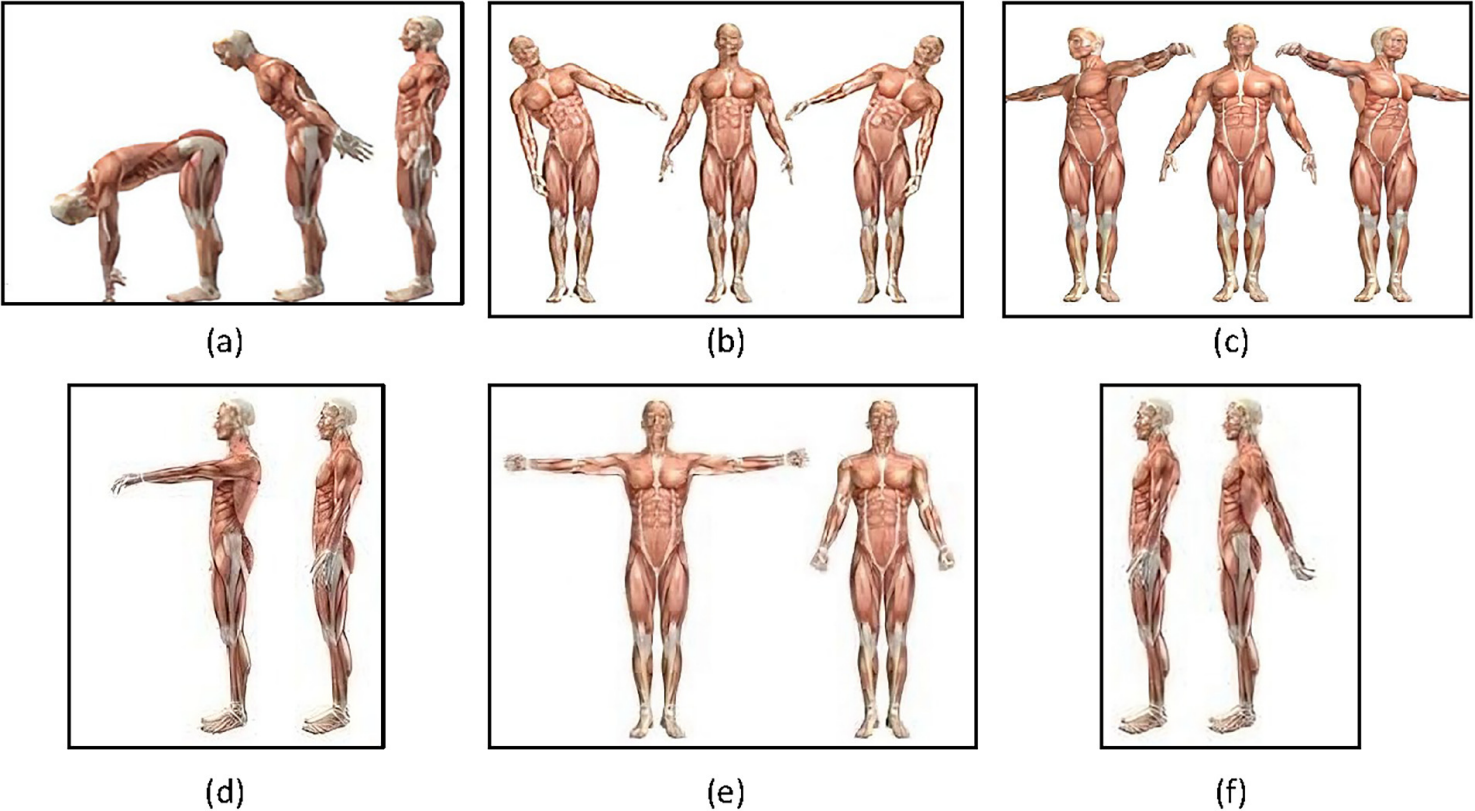
Motor task for spine and shoulder active ROM assessment. Anterior flexion **(a)**; lateral bending **(b)**; twist **(c)**; frontal rise **(d)**; lateral rise **(e)**; and backward push **(f).**

Each participant performed a series of six tasks designed to evaluate the active ROM of the spine and shoulders. Starting from an upright standing position with arms extended and forearms aligned, participants performed the following motor tasks (Figure 3):

• Anterior trunk flexion: participants bent forward at the waist while keeping the spine as neutral as possible, aiming to reach toward the ground or shins without bending the knees. This task measured spinal flexion (Figure 3a);
• Lateral bending (right/left): participants bent the torso sideways at the waist, bringing one arm toward the leg while maintaining hip stability. This task measured spinal lateral flexion (Figure 3b);
• Twist (right/left): participants rotated the torso to one side, while keeping the hips facing forwards. This task measured spinal rotation (Figure 3c);
• Frontal arm rise (right/left): participants raised the arm straight in front of the body to the maximum achievable height while keeping the elbow straight. This task measured shoulder flexion (Figure 3d);
• Lateral arm rise (shoulder abduction) (right/left): participants raised the arm laterally out to the side to the maximum achievable height while maintaining a straight elbow. This task measured shoulder abduction (Figure 3e);
• Backward arm push (shoulder extension) (right/left): participants extended the arm backwards behind the body, maintaining a straight elbow throughout the movement. This task measured shoulder extension (Figure 3f).

Each task was performed in two sets of six repetitions (per side for bilateral movements). Participants were instructed to perform the movements at their natural, self-selected pace, within their maximum pain-free active ROM.

**Figure 4:**
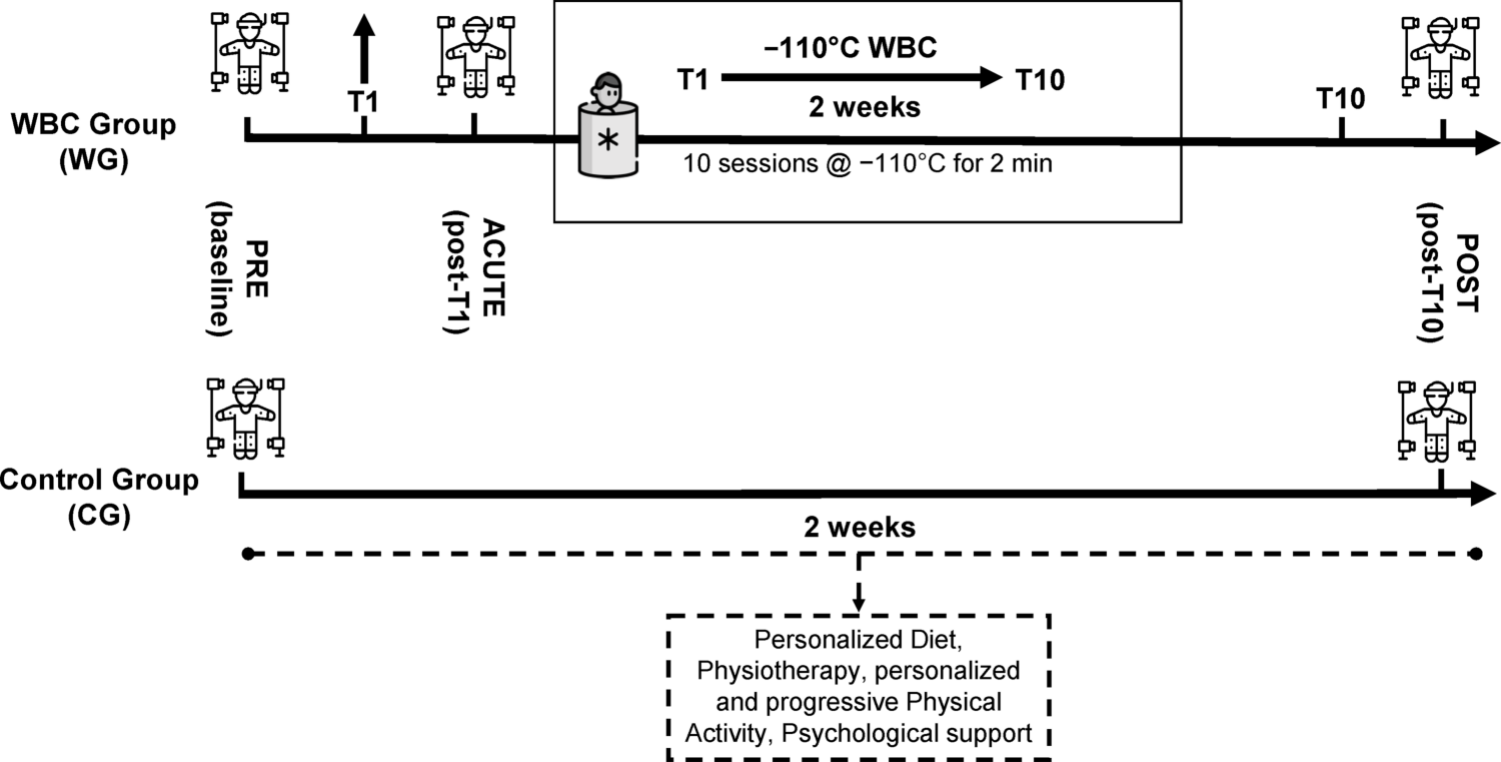
Graphical representation of the treatment protocol.

The assessment was conducted at distinct time points to quantitatively evaluate the effects of the rehabilitation programs with and without WBC on WG and CG. Specifically, WG was evaluated at baseline (i.e., PRE), immediately after the first WBC session (i.e., ACUTE), and at the completion of the WBC cycle (i.e., POST). In contrast, CG was assessed at baseline (i.e., PRE) and following a rehabilitation period matched in duration to the WBC cycle (i.e., POST). A graphical representation of the treatment protocol and its timeline is provided in Figure 4.

### 2.4 Data analysis and processing

Raw optical data were initially processed using VICON Nexus software (version 1.8., Vicon, Oxford Metrics Ltd., Oxford, UK). During this process, each marker was assigned to its corresponding anatomical landmark. Processed data were then imported into SmartAnalyzer (BTSBioengineering, Milan, Italy) for further analysis with custom routines to extract quantitative measurements.

The 3D marker coordinates were first linearly interpolated and filtered using a 5 Hz low-pass Butterworth filter to reduce noise. To ensure accurate angle measurements and minimize alignment errors between the participant's body and laboratory reference system (X, Y, Z), a local reference frame (x, y, z) was defined on the pelvis, following the method described by Menegoni et al. (63). Specifically:

• The *x*-axis (medial-lateral) was aligned with the markers on the right and left anterior superior iliac spine (RASIS and LASIS), and oriented from the left to the right of the subject.
• The *y*-axis (vertical) was defined as the perpendicular to the pelvic plane (i.e., plane formed by the vector connecting RASI to RPSI), and directed upwards.
• The *z*-axis (anterior-posterior) was defined as the cross product of the x- and y-axes.

Task-specific routines were then applied to calculate relevant rotation angles and their corresponding ROMs. Repetitions for each task were identified by detecting the maximum and the minimum values along the angular curve, focusing on the primary rotation angles associated with each movement (i.e., shoulder flexion in frontal arm rise). For each repetition, ROM was computed as the difference in degrees (°) between the maximum and the minimum. Additionally, task duration (seconds) was calculated as the difference between the end of the last repetition and the start of the first repetition. The analyzed angles and their definitions are detailed in Table 2.

**Table 2:** Definitions of biomechanical angles for shoulder and spinal movements.

Task	Measured angle	Angle label	Definition
Frontal rise	Shoulder flexion	SF	Angle defined by anterior elevation of the arm relative to the torso
Lateral rise	Shoulder abduction	SA	Angle defined by the lateral elevation of the arm relative to the torso
Backward push	Shoulder extension	SE	Angle defined by the posterior displacement of the arm relative to the torso
Lateral bending	Spinal inclination (Absolute)	SIA	Angle between the vector passing through LACR and RACR and the Y-axis
	Spinal inclination (Local)	SIL	Angle between the vector passing through LACR and RACR and the y-axis
Twist	Spinal rotation (Absolute)	SRA	Angle measured between the vector passing through LACR and RACR and the Z-axis.
	Spinal rotation (Local)	SRL	Angle measured in the coronal plane between the vector passing through LACR and RACR and the z-axis.
Anterior flexion	Lower spine inclination	S1-L1	Angle of the lower spinal segment (S1-L1) with respect to Y-axis
	Mid-lower spine inclination	S1-L3	Angle of the mid-lower spinal segment (S1-L3) with respect Y-axis
	Mid spine inclination	L1-T1	Angle of the mid spinal segment (L1-T1) with respect to the Y-axis.
	Upper-mid spine inclination	L1-T6	Angle of the upper-mid spinal segment (L1-T6) with respect to Y-axis
	Upper spine inclination	T6-T1	Angle of the upper spinal segment (T6-T1) with respect to Y-axis
	Pelvic inclination	Pelvis	Angle between the projection of the S1-L1 vector and y-axis
	Trunk inclination	Trunk	Angle between the projection of the S1-T1 vector and the Y-axis
	Thoracic inclination	Thorax	Angle between the projection on the sagittal plane of vector T1-L1 and vector S1-L1

Movements are categorized by tasks, measured angles, labels, and their definitions. Spinal movements are described in relation to both local (x, y, z) and laboratory (X, Y, Z) reference frame as well as by specific spinal segment. Shoulder movements are categorized based on sagittal and frontal plane actions.

### 2.5 Statistical analysis

Statistical analysis was performed with JASP (JASP Team, Amsterdam, Netherlands) and Matlab (MathWorks Inc., Natick, MA, United States). Normality of data was assessed with the Shapiro–Wilk test, while homogeneity of variances was verified using Levene's test. A preliminary paired *t*-test was performed to compare the right and left sides during bilaterally executed tasks. Independent *t*-tests were conducted at baseline to evaluate potential group differences in anthropometric measures and performance parameters.

To assess whether changes over time differed between groups, a mixed repeated measures ANOVA (mixed RM-ANOVA) was performed using only the PRE and POST data. This analysis aimed to examine the interaction between time (i.e., session) and group (i.e., WG vs. CG), with a significant session*group interaction (*p* < 0.05) indicating that the time effect on the outcome variable was not the same for both groups.

Within WG, RM-ANOVA was performed across all the available sessions (i.e., PRE, ACUTE, and POST) to evaluate temporal changes in ROM and task duration. The inclusion of the ACUTE time point allowed for a more comprehensive evaluation of the ROM progression over time, which was not captured by the mixed RM-ANOVA that only included the PRE and POST sessions. If a significant main effect of session was found (*p* < 0.05), *post hoc* analysis were conducted using multiple *t*-test comparisons with Holm correction to identify significant differences between specific time points (i.e., PRE vs. POST, PRE vs. ACUTE, ACUTE vs. POST). Effect sizes for *post hoc* comparisons were reported as Cohen's *d* (*d*) and interpreted as follows: *d* < 0.20, small effect; 0.20 ≤ *d* < 0.50, moderate effect; 0.50 ≤ *d* < 0.80, large effect; and *d* ≥ 0.80, very large effect. For CG, paired *t*-tests were applied to detect PRE-POST changes, as data were available only for these two sessions according to the study design.

Finally, an additional analysis was performed to evaluate the onset of fatigue during the tasks involving spinal movements. ROM is influenced by the shortening capacity of muscles across a joint, which can be diminished by fatigue. Fatigue, in turn, reduces the ability of muscles to shorten effectively, leading to a reduction in joint ROM during motor task (65). In this study, the presence of fatigue was assessed using RM-ANOVA to examine changes across the performed repetitions in terms of ROM for each session, followed by *post hoc* tests to identify specific differences between repetitions if needed.

## 3 Results

The Shapiro–Wilk test confirmed the normality of data distribution, while Levene's test verified the homogeneity of variances across all groups and tasks for all parameters, supporting their representation in terms of mean and standard deviation. Preliminary paired *t*-tests revealed no statistically significant differences (*p* > 0.05) in baseline parameters between groups. Similarly, no statistically significant differences (*p* > 0.05) were found between the right and left sides for bilaterally performed tasks. Based on these results, data from both sides were pooled, and ROM was calculated as the average across repetitions per each set, excluding the first repetition per side to minimize potential initial measurement biases (64, 66). Finally, the average ROM for each participant was computed as the mean value from the two sets.

### 3.1 Skin temperature measurements

A significant reduction in skin temperature was consistently observed following WBC sessions, providing direct evidence of the treatment's potent cooling effect. Table 3 reports the mean percentage changes in temperature recorded before and after the first treatment and before and after the final treatment for subjects allocated to the WG group.

**Table 3:** Mean percentage changes (Δ%) and mean temperature drops (ΔT), reported as mean (SD), between pre-T1 and post-T1 and pre-T10 and post-T10 WBC sessions at four reference points in WG (*n* = 21): nape (N), quadriceps (Q), popliteal cavity (PC), and calf (C).

Reference point	Mean Δ% - T1	Mean ΔT°C - T1	Mean Δ% - T10	Mean ΔT°C - T10
Nape	−17.90 (8.19)	−4.44 (3.46)	−20.01 (9.09)	−5.04 (3.90)
Quadriceps	−30.46 (9.37)	−7.19 (4.86)	−36.64 (13.20)	−8.60 (5.97)
Popliteal cavity	−43.73 (12.55)	**−**10.46 (6.82)	−46.35 (15.26)	−11.06 (7.39)
Calf	−46.26 (13.28)	−11.11 (7.31)	−45.54 (12.98)	−10.87 (7.06)

### 3.2 Rehabilitation and treatment effects across sessions

#### 3.2.1 Shoulder

**Figure 5:**
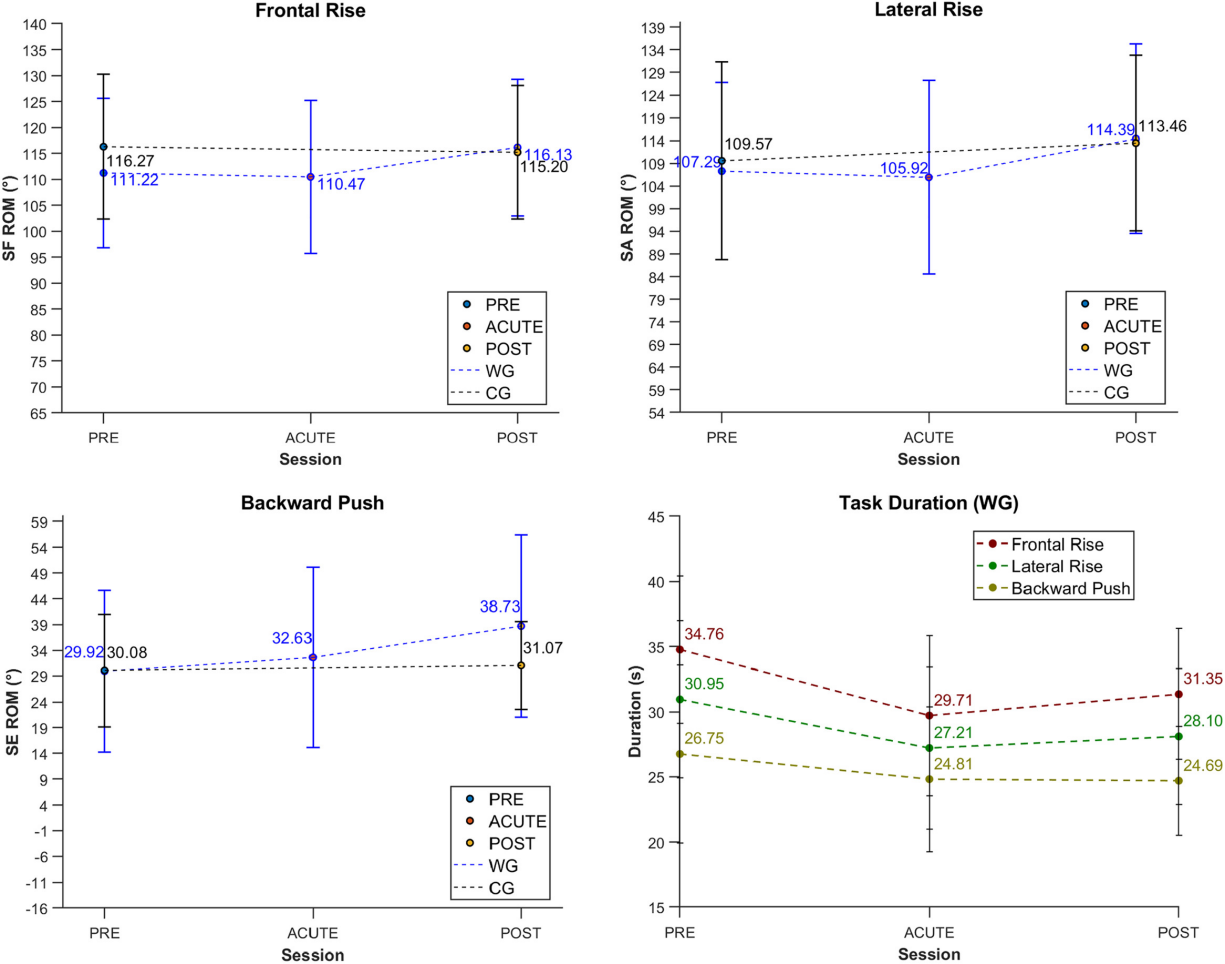
Visual representation of the mean values and standard deviations of ROM and duration for shoulder motor tasks across sessions and groups. ROM values for the WG are displayed in blue, while those for the CG are shown in black.

Table 4 summarizes the mean ROM and task duration for shoulder-related motor tasks across sessions, divided by group, along with the corresponding statistical analysis results. These results are also visually displayed in Figure 5. For conciseness, the complete results of the statistical analysis, including the full set of *p*-values and effect size, are provided in the Supplementary Material.

**Table 4:** Mean and standard deviation for shoulder motor assessment across the different sessions, in terms of ROM and duration.

Task	Parameter	WG			CG	
		PRE	ACUTE	POST	PRE	POST
Frontal rise	SF ROM (°)	111.22 (14.38)	110.47 (14.76)	116.13 (13.18)**^+^**	116.27 (13.95)	115.20 (12.86)
	Duration (s)	34.76 (5.66)	29.71 (6.13)**^†^**	31.35 (5.03)*	36.18 (6.93)	34.68 (8.00)
Lateral rise	SA ROM (°)	107.29 (19.52)	105.92 (21.33)	114.39 (20.86)**^+^**	109.57 (21.76)	113.46 (19.36)
	Duration (s)	30.95 (6.05)	27.21 (6.21)**^†^**	28.10 (5.19)*	32.21 (7.41)	30.55 (7.77)
Backward push	SE ROM (°)	29.92 (15.67)	32.63 (17.54)	38.73 (17.70)*^,+^	30.08 (10.94)	31.07 (8.55)
	Duration (s)	26.75 (6.82)	24.81 (5.56)^†^	24.69 (4.18)*	27.22 (4.95)	25.40 (4.99)

Assessments were conducted at three time points for WG: PRE (i.e., baseline), ACUTE (i.e., post-T1) and POST (i.e., post-T10). For CG, assessment was conducted at PRE and POST only. Statistical significance for within-group comparisons is indicated as follows: WG: “*” = *p* < 0.05 if PRE vs. POST; “†” = *p* < 0.05 if PRE vs. ACUTE; “+” = *p* < 0.05 if ACUTE vs. POST.

The mixed RM-ANOVA revealed a significant session*group interaction for SE ROM during the backward push task (*p* = 0.003), indicating group-specific improvement from PRE to POST in WG. However, no significant interactions were found for the other tasks.

With respect to the temporal evolution of ROM and task duration within WG, RM-ANOVA revealed statistically significant differences over time for both ROM and task duration. In particular, *post hoc* tests showed that SF ROM significantly increased from ACUTE to POST in the frontal rise task (*p* = 0.045), while in the lateral rise task, SA ROM significantly increased from PRE to ACUTE (*p* = 0.036). Similarly, SE ROM showed significant improvements from ACUTE to POST (*p* = 0.002), as well as from PRE to POST (*p* < 0.001). Task duration significantly decreased across all tasks between PRE and ACUTE, as well as between PRE and POST (*p* < 0.05).

*post hoc* comparisons also indicated that significant differences (*p* < 0.05) were associated with moderate (0.20 ≤ *d* < 0.50) to large (0.50 ≤ *d* < 0.80) effect sizes, supporting the impact of WBC over time. Conversely, non-significant comparisons (*p* > 0.05) showed small effect sizes (*d* < 0.2), pointing to minimal or negligible differences between the observed time points.

In contrast, CG did not show statistically significant changes in terms of ROM or duration between PRE and POST sessions for any tasks.

#### 3.2.2 Spine

**Figure 6:**
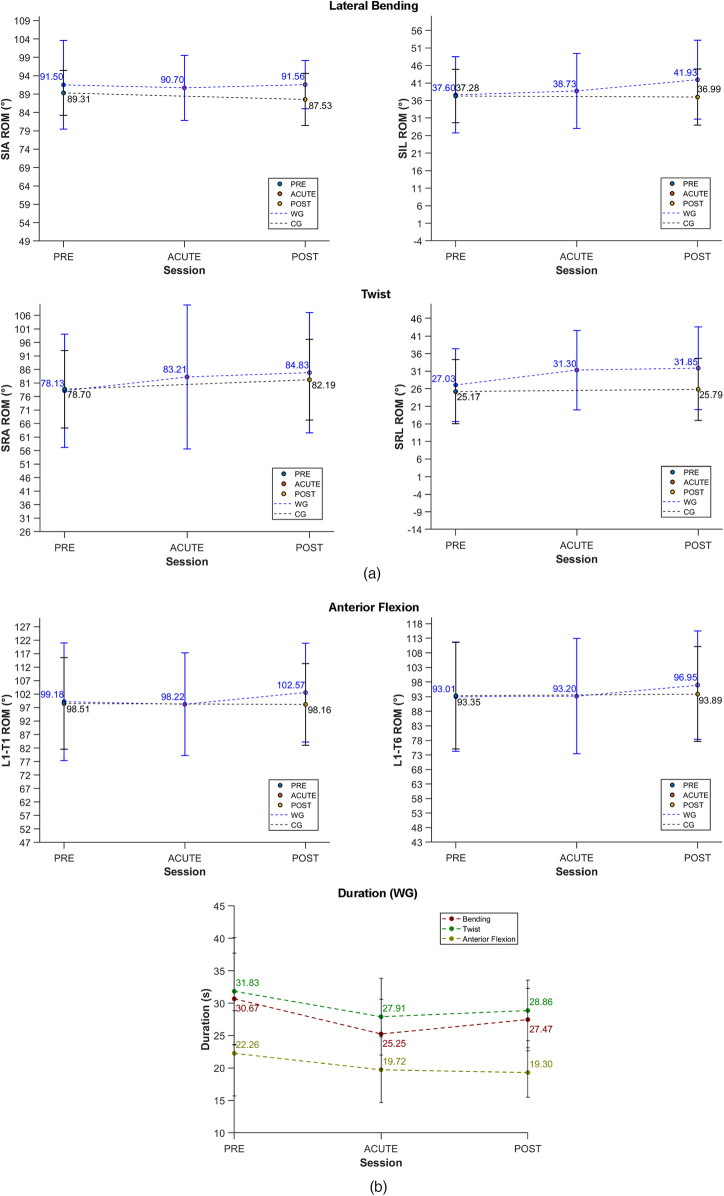
Visual representation of the mean values and standard deviations of ROM and duration for tasks involving spinal movements across sessions and groups. ROM values for the WBC are displayed in blue, while those for the CG are shown in black. Lateral bending and twist task **(a)** Anterior flexion and task durations **(b).**

The results for motor tasks targeting spinal movements are summarized in Table 5 and Figure 6, following the same format as the shoulder analysis in the previous section.

**Table 5:** Mean and standard deviation for spinal motor assessment across the different sessions, in terms of ROM and duration.

Task	Parameter	WG			CG	
		PRE	ACUTE	POST	PRE	POST
Lateral bending	SIL ROM (°)	37.60 (10.88)	38.73 (10.67)	41.93 (11.26)*^,+^	37.28 (7.59)	36.99 (7.99)
	SIA ROM (°)	91.50 (12.06)	90.70 (8.85)	91.56 (6.57)	89.31 (9.33)	87.53 (6.11)
	Duration (s)	30.67 (7.05)	25.25 (5.38)^†^	27.47 (4.81)*	34.66 (7.10)	30.83 (5.13)*
Twist	SRL ROM (°)	27.03 (10.32)	31.30 (11.29)†	31.85 (11.74)*	25.17 (9.09)	25.79 (8.81)
	SRA ROM (°)	78.13 (20.96)	83.21 (26.66)	84.83 (22.25)	78.70 (14.33)	82.19 (14.94)
	Duration (s)	31.83 (8.28)	27.91 (5.93)†	28.86 (4.68)*	34.51 (8.10)	32.35 (5.59)
Anterior flexion	S1-L1 ROM (°)	69.70 (16.48)	70.66 (17.31)	66.45 (17.27)	63.00 (17.36)	63.66 (14.62)
	S1-L3 ROM (°)	56.32 (15.87)	54.94 (16.31)	50.85 (15.84)	50.55 (15.84)	50.57 (14.28)
	L1-T1 ROM (°)	99.18 (21.82)	98.22 (19.04)	102.57 (18.32)	98.51 (16.96)	98.16 (15.13)
	L1-T6 ROM (°)	93.01 (18.75)	93.20 (19.77)	96.95 (18.59)	93.35 (18.24)	93.89 (16.27)
	T6-T1 ROM (°)	101.77 (22.78)	99.96 (19.63)	103.58 (17.38)	101.30 (13.96)	100.54 (14.74)
	Pelvis ROM (°)	31.33 (19.28)	29.51 (16.00)	28.47 (15.73)	21.42 (12.79)	25.54 (13.53)
	Trunk ROM (°)	88.52 (17.53)	88.25 (17.53)	90.84 (16.16)	86.71 (15.77)	87.32 (15.23)
	Thorax ROM (°)	32.31 (12.00)	29.17 (10.72)	37.17 (15.18)+	38.00 (11.80)	35.51 (11.99)
	Duration (s)	22.26 (6.57)	19.72 (5.07) †	19.30 (3.82)*	25.54 (5.69)	22.77 (3.80)*

Assessments were conducted at three time points for WG: PRE (i.e., baseline), ACUTE (i.e., post-T1) and POST (i.e., post-T10). For CG, assessments were conducted at PRE and POST only. Statistical significance for within-group comparisons is indicated as follows: WG: “*” = *p* < 0.05 if PRE vs. POST; “†” = *p* < 0.05 if PRE vs. ACUTE; “+” = *p* < 0.05 if ACUTE vs. POST; CG group: “*” = *p* < 0.05 if PRE vs. POST.

The mixed RM-ANOVA revealed significant session*group interactions (*p* < 0.05) for several parameters. Specifically, significant interactions were found for SIL ROM in the bending task (*p* = 0.014), SRL ROM (*p* = 0.048), and task duration across all tasks. These interactions suggest that the improvements in ROM and reductions in task duration over time were significantly greater in WG compared to CG.

For the bending tasks, RM-ANOVA revealed that SIL ROM significantly increased from ACUTE to POST (*p* = 0.035) and from PRE to POST (*p* = 0.005), with a total improvement of about 4°. In the twist task, significant differences were observed from PRE to POST for SRL ROM (*p* = 0.029), which increased by about 5°. For anterior flexion, although WG showed trends toward ROM improvements, no significant differences were found (all *p* > 0.05). For instance, L1-T6 ROM and T6-T1 ROM showed variations, but neither reached statistical significance (*p* = 0.095 and *p* = 0.397, respectively). In addition, a significant decrease in duration was observed for all tasks from PRE to ACUTE and from PRE to POST. As seen with shoulder movements, *post hoc* comparisons in WG following RM-ANOVA indicated moderate (0.20 ≤ *d* < 0.50) to large (0.50 ≤ *d* < 0.80) effect sizes for significant comparisons, supporting a sustained impact of the treatment over time.

In contrast, CG did not report statistically significant changes in ROM or duration between PRE and POST sessions for any tasks. However, a significant reduction (*p* = 0.01) in task duration was observed from PRE to POST for bending and anterior flexion.

### 3.3 Fatigue onset and effects of WBC

**Figure 7:**
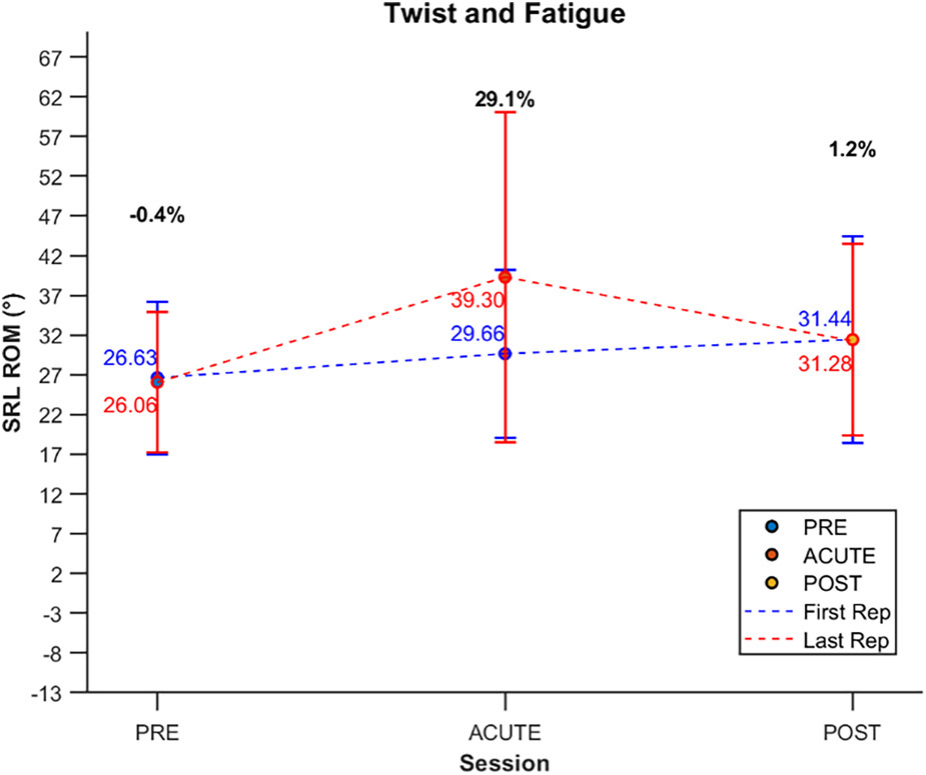
Visual representation of mean values and standard deviations for the first and last repetitions for SRL ROM during the twist task in WG across PRE, ACUTE, and POST sessions. Data points for the first repetition are shown in blue, while those for the last repetition are shown in red. Percentage change between the first and the last repetitions is indicated in bold black on the graph for each session.

No significant fatigue onset was detected for the bending and anterior flexion tasks, where the ROM remained almost stable across the repetitions performed in each session. However, for the twist task, significant differences were observed in SRL ROM between the first considered repetition and the last repetition. Notably, these findings do not seem to indicate fatigue onset but rather a significant enhancement in performance. In fact, while ROM remained consistent across repetitions during the PRE and POST sessions, a progressive increase in ROM was observed from the initial to the last repetition in the ACUTE session. This pattern seems to suggest immediate improvement following WBC treatment, particularly for rotational movements. The absence of significant changes during the PRE and POST sessions further supports the notion that the observed enhancement is specific to the acute effects of WBC. These results are presented in Table 6 and Figure 7.

**Table 6:** Mean values and standard deviations for SRL ROM during the twist task in WG are presented for the first and last repetitions across PRE, ACUTE, and POST sessions.

Session	ROM first rep (°)	ROM last rep (°)	% Change
PRE	26.63 (9.58)	26.06 (8.81)	−0.4%
ACUTE	29.66 (10.59)	39.30 (20.73)*	+29.1%
POST	31.44 (12.98)	31.28 (12.06)	+1.2%

The percentage change (% Change) between the first and last repetitions is reported for each session.

* *p* < 0.05 if first rep vs. last rep.

## 4 Discussion

Reduced ROM, particularly at shoulder and spine level, is common in individuals with obesity, leading to pain, functional impairments and decreased quality of life. WBC, which involves controlled exposure to extremely low temperatures (typically between −110°C and −140°C) for short durations, has been shown to reduce inflammation, pain, and oxidative stress. In our study, we consistently observed a significant reduction in skin temperature following WBC sessions, providing direct evidence of the treatment's potent cooling effect, which is thought to initiate a cascade of physiological responses. The exposure to cold temperatures has been shown to induce systemic physiological responses, such as vasoconstriction followed by vasodilation upon rewarming, promoting enhanced circulation and metabolic recovery (38). This process is thought to decrease inflammatory markers and improve muscle recovery, which could potentially impact mobility (15, 30). In this context, the aim of this original research was to investigate the impact of WBC combined with physiotherapy interventions on active ROM in individuals with obesity, using a 3D marker-based optoelectronic MoCap system, the gold standard for human motion analysis and functional assessment. By tracking these changes within rehabilitation, this study aimed to provide quantitative insights into how WBC-related mechanisms may impact mobility in this population.

The results from 42 patients with a chronic, remitting and relapsing condition like obesity shows evidence that repeated WBC sessions combined with a rehabilitation program can significantly improve spinal and shoulder active ROM as compared to rehabilitation alone. The findings offer quantitative insights into both the acute and short-term effects of WBC, supporting its potential as an effective therapeutic intervention for enhancing flexibility and ROM of the spine and shoulders, thus managing some of the obesity-related musculoskeletal limitations. Although we did not directly measure weight changes during the initial two-week intervention, the comparable and minimal weight loss observed in both the WBC and control groups at the end of the rehabilitation period, especially when considered in relation to the participants' initial weight, suggests that substantial weight fluctuations during the intervention are unlikely to be the main factor in the observed ROM improvements. Given this similar and modest weight reduction in both groups, it becomes less likely that changes in body mass contributed significantly to the improvement in range of motion, thus strengthening the argument that the ROM improvements are primarily attributable to the WBC intervention.

### 4.1 Acute effects

Changes in active ROM after the first WBC session were observed: they were not significant in shoulder flexion and abduction, whereas in spinal rotation a significant (*p* < 0.05) increase was observed (PRE, Mean: 27.03°, SD: 10.32; ACUTE, Mean: 31.85°, SD: 11.74°). An immediate benefit of WBC was evident after just one session, as significant reductions in task duration were observed across all tasks. For instance, the duration of the frontal raise task decreased from pre-intervention (PRE, Mean: 34.76 s, SD: 5.66 s) to post-WBC (ACUTE, Mean: 29.71 s, SD: 6.13 s). Similarly, the twist task duration dropped from the same time points (PRE, Mean: 31.83 s, SD: 8.28 s; ACUTE, Mean: 27.91 s, SD: 5.93 s). It is worth noting that De Nardi et al. (57) also demonstrated significant improvements in sit-and-reach ROM after a single WBC session, highlighting the potential for acute ROM enhancements with this intervention. However, in our study, we observed that these acute ROM improvements were specific to spinal rotation, while task duration was reduced across all movements. We can hypothesize that these rapid improvements are due to the synergic local and systemic effects of WBC previously described, reducing discomfort during movement even after a single session. The observation of these acute improvements in spinal rotation and task durations specifically in the WBC group, unlike the control group, further supports the notion that the enhanced ROM is a direct effect of the WBC treatment and not a consequence of weight loss occurring over the rehabilitation period.

### 4.2 Post-cycle effects

After the 10-session WBC cycle, significant (*p* < 0.05) improvements in active ROM were observed in spinal and shoulder movements, with the magnitude of improvement varying depending on the task and anatomical region. Shoulder active ROM increased significantly in all tasks, especially during the backward push task (PRE, Mean: 29.92°, SD: 15.67°; POST, Mean: 38.73°, SD: 17.70°). Similarly, active spinal ROM showed significant gains in spinal lateral bending and rotation, but not in anterior flexion. Task durations decreased significantly in all tasks, with further improvements compared to the acute changes, suggesting that WBC's impact on neuromuscular efficiency and coordination is consistent regardless of the movement type. Our results also suggest that while WBC rapidly alleviates muscle stiffness and discomfort, its effects on active ROM may require multiple sessions to achieve substantial improvements.

### 4.3 Fatigue onset and task-specific insights

Novel insights were observed in fatigue onset, particularly during spinal rotation in the twist task, where a progressive increase in SRL ROM was reported from the first to the last repetition during the acute assessment. This contrasts with the typical fatigue-related decline observed in repetitive motor tasks. Enhanced proprioception and sensory feedback, combined with neurovascular changes such as increased blood flow and oxygenation, likely contributed to this phenomenon. In contrast, the PRE session showed no significant intra-session changes in ROM, indicating baseline capacity without external facilitation. By the POST session, participants demonstrated short-term adaptations, including improved baseline spinal mobility and neuromuscular efficiency, minimizing the need for acute facilitatory effects.

### 4.4 Underlying mechanisms

The observed benefits in shoulder and spinal active ROM can be attributed to the multifaceted physiological mechanisms elicited by WBC. Firstly, cryotherapy may reduce pain and inflammation by regulating the release of anti-inflammatory cytokines and endorphins, which may accelerate tissue recovery and enhance mobility (67). Secondly, the alternating vasoconstriction and vasodilation cycle may improve blood circulation and oxygen delivery to tissues, potentially relieving stiffness and enabling smoother movement (68). Additionally, the findings suggest that WBC may positively influence neuromuscular control and proprioceptive feedback, potentially contributing to optimized motor performance and reduced discomfort during physical tasks. A systematic review (69) examined 11 trials investigating the effects of local cryotherapy in flexibility. Based on those findings, it appears that there is some credible research evidence supporting the notion that cryotherapy can enhance joint flexibility. Certain researchers support the theory that cryotherapy positively affects the viscoelastic properties of a muscle unit (70) and the muscle myostatic reflex (71). These two mechanisms can contribute to greater muscle relaxation and, thus, to higher flexibility. Further, cryotherapy for a stretched muscle has been found to cause depression of stretch reflex through direct sensory stimulation of the primary and secondary muscle spindle afferent fibers, which decrease activity and lower a muscle's threshold to interfere in muscle excitability (72). In addition, localized cryotherapy, including ice application, has been shown to decrease nerve conduction velocity and muscle spasms (73) and affect muscle properties and neural activity (74). However, as demonstrated by Harlaar et al. (74), these effects do not consistently translate to significant improvements in ROM, particularly in patients with spasticity. These combined effects are particularly significant for individuals with obesity, who often experience restricted ROM, joint pain, and early onset of fatigue. By addressing these limitations, WBC has shown to improve the active ROM in spine and shoulder. Our systemic WBC approach resulted in more substantial gains in spinal and shoulder ROM, suggesting that WBC may enhance joint mobility, although the underlying physiological mechanisms, involving both local and systemic effects, remain unclear and were not specifically investigated in this study. Notably, the improvements observed in WG were absent in CG, where active ROM and task duration exhibited minimal or non-significant changes, reinforcing the hypothesis that the observed benefits are attributable to WBC. The patient's positive emotional engagement with the treatment and satisfaction with a brief, well-tolerated procedure without side effects may have played a role in the observed functional improvements and a placebo effect cannot be ruled out.

### 4.5 Statistical insights and temporal evolution of ROM

Despite the small sample size, the moderate-to-large effect sizes for significant differences in *post hoc* tests following RM-ANOVA suggest a sustained impact of WBC over time. These effect sizes indicate that the observed improvements are not only statistically significant but also meaningful in real-world terms, implying functional benefits and emphasizing the potential clinical relevance of the treatment to achieve meaningful mobility improvements.

The mixed RM-ANOVA, which assessed the interaction between time (i.e., session) and group (i.e., WG vs. CG), was performed using only the PRE and POST sessions. This analysis revealed significant interactions for several parameters (e.g., SIL ROM and SRL ROM). However, to examine the evolution of ROM across all time points for WG (i.e., PRE, ACUTE, and POST), a separate RM-ANOVA was conducted. This analysis was specifically intended to assess changes in ROM and task duration over time for WG, even when no significant interaction was found between PRE and POST in the mixed-design ANOVA. By including the ACUTE time point, a more comprehensive progression of ROM was observed, which was not captured by the mixed ANOVA focusing only on PRE and POST.

While the mixed RM-ANOVA did not reveal significant interactions between group and time for PRE and POST for certain parameters (e.g., SF ROM), this does not imply that the WG group did not experience substantial changes. For this reason, within-group analysis across all time points were important to fully capture the evolution of ROM over the entire treatment period, including the acute phase, where initial changes may have occurred.

This dual approach enhances the understanding of the temporal effects of WBC and supports its potential as a clinically relevant intervention for improving mobility, further emphasizing the importance of considering multiple time points in future research.

### 4.6 Strengths and limitations

A significant strength of this study is the use of a MoCap system to quantitively assess the effect of WBC on mobility parameters. MoCap systems are widely regarded as the gold standard for human motion analysis, with excellent accuracy and reliability demonstrated in prior research. Unlike previous studies relying on subjective measures or manual tools like goniometers, MoCap provided precise and reproducible data on movement patterns and changes. The adoption of a marker placement protocol specifically developed for individuals with obesity further enhanced data accuracy, addressing concerns related to adiposity and altered body geometry. This methodological rigor sets a new standard for evaluating therapeutic interventions in this population. Another strength of the study is the use of WBC instead of local cryotherapy. Using a whole-body cold exposure may in fact amplify the results that can be obtained with local cold therapies, taking advantage of the cascade of systemic effects that have been previously documented after WBC.

However, several limitations that deserve consideration are present. Firstly, a consecutive enrollment approach was used, rather than randomization, because of clinical and logistical constraints. Conducted in the hospital setting, randomization proved impractical for maintaining patient tolerance and adherence to the intervention, reflecting clinical practice in which treatment decisions prioritize patient well-being and practical feasibility. Furthermore, the absence of a crossover design is due to the patients' limited rehabilitation period and hospital stay, which made it impractical to implement within the study time frame. As for a placebo-controlled design, patients would have easily detected the lack of steam and cold inside the chamber, compromising the integrity of the placebo effect, especially in later sessions. Additionally, the scope of the study was limited by the absence of multiple outcome measures, such as cytokines and other inflammatory parameters. This decision was made because the small sample size would likely have precluded the detection of significant changes between groups, since these parameters often improve during the rehabilitation process. A larger cohort would be required for such an analysis. Regarding additional assessments of ROM, the patients' tight rehabilitation timeframes necessitated a focused assessment on those measures deemed most relevant to our study objectives. In addition, the relatively small sample size and short-term focus limit the generalizability of the findings. Future research should explore WBC benefits in the long-term and its efficacy across diverse populations. Additionally, the task-specific nature of improvements warrants further investigation to optimize cryotherapy protocols for various movement patterns and functional needs.

## 5 Conclusions

This study highlights the significant benefits of adding WBC to rehabilitation programs in enhancing spinal and shoulder active ROM in individuals with obesity. The acute effects of WBC provide rapid increase of ROM, while the benefits observed after 10 repeated sessions underscore the added value of a 10-session WBC cycle for achieving clinically meaningful functional outcomes. The consistent decrease in skin temperature observed after each WBC session supports the treatment's effective cooling properties, likely triggering a cascade of physiologic responses that contribute to these positive improvements. In particular, a considerable reduction in skin temperature from before to after the session was observed. This suggests that WBC may elicit beneficial effects through alternative mechanisms or that even a less pronounced reduction in temperature may produce clinically relevant results in this population.

By increasing active ROM, reducing task duration, and enhancing neuromuscular efficiency, WBC offers a versatile therapeutic approach to address obesity-related mobility challenges and improving overall quality of life. This treatment has proven to be safe once medical screening has been performed (75). Although sufficient evidence suggesting an improved joint flexibility after local cryotherapy in athletes has been shown (69), future randomized controlled trials on larger sample sizes of patients with ROM limitations using gender-specific analyses, and extended follow-up periods are needed to validate these findings further and to implement add-on cold therapies to boost rehabilitation outcomes.

## Data Availability

The datasets presented in this study can be found in online repositories. The names of the repository/repositories and accession number(s) can be found below: Zenodo (https://doi.org/10.5281/zenodo.14735132).
