# Supplementary material for: Effects of whole-body cryostimulation on spinal and shoulder range of motion in individuals with obesity
Source: Front Rehabil Sci. 2025 Jul 23;6:1568280. doi: 10.3389/fresc.2025.1568280 (PMC12325352; doi:10.3389/fresc.2025.1568280)
Supplement: Supplementary file 1 [file Datasheet1.pdf]

Supplementary Material

Supplementary Table 1. Full results of the statistical analysis. Statistical tests include repeated measures ANOVA (RM-ANOVA) for the WG group, paired t-tests for the CG group, and mixed RM-ANOVA to assess the Time × Group interaction. Post-hoc comparisons following RM-ANOVA were conducted using multiple t-tests with Holm correction. Effect sizes are reported as Cohen's d for post-hoc comparisons following RM-ANOVA.

Task	Parameter	Group	Session	Mean (SD)	Statistical Test	p-value	Post-hoc Comparison	Post-hoc p-value	Effect Size (d)
Frontal Rise	SF ROM (°)	WG	PRE	111.22 (14.38)	RM-ANOVA	0.034	PRE vs ACUTE	0.725	0.06
			ACUTE	110.47 (14.76)			ACUTE vs POST	0.045	0.43
			POST	116.13 (13.18)			PRE vs POST	0.075	0.37
		CG	PRE	116.27 (13.95)	Paired t-test	0.56	-	-	-
			POST	115.20 (12.86)			-	-	-
		Interaction	Session*Group	-	Mixed RM-ANOVA	0.088	-	-	-
	Duration (s)	WG	PRE	34.76 (5.66)	RM-ANOVA	< 0.001	PRE vs ACUTE	< 0.001	0.88
			ACUTE	29.71 (6.13)			PRE vs POST	0.186	0.29
			POST	31.35 (5.03)			ACUTE vs POST	0.016	0.59
		CG	PRE	36.18 (6.93)	Paired t-test	0.33	-	-	-
			POST	34.68 (8.00)			-	-	-
		Interaction	Session*Group	-	Mixed RM-ANOVA	0.351	-	-	-
Lateral Rise	SA ROM (°)	WG	PRE	107.29 (19.52)	RM-ANOVA	0.044	PRE vs ACUTE	0.561	0.10
			ACUTE	105.92 (21.33)			ACUTE vs POST	0.036	0.44
			POST	114.39 (20.86)			PRE vs POST	0.094	0.35
		CG	PRE	109.57 (21.76)	Paired t-test	0.28	-	-	-

			POST	113.46 (19.36)			-	-	-
		**Interaction**	Session*Group	-	Mixed RM-ANOVA	0.455	-	-	-
	**Duration (s)**	**WG**	PRE	30.95 (6.05)	RM-ANOVA	0.007	PRE vs ACUTE	0.003	0.63
			ACUTE	27.21 (6.21)			PRE vs POST	0.400	0.15
			POST	28.10 (5.19)			ACUTE vs POST	0.019	0.48
		**CG**	PRE	32.21 (7.41)	Paired t-test	0.26	-	-	-
			POST	30.55 (7.77)			-	-	-
		**Interaction**	Session*Group	-	Mixed RM-ANOVA	0.525	-	-	-
**Backward Push**	**SE ROM (°)**	**WG**	PRE	29.92 (15.67)	RM-ANOVA	< 0.001	PRE vs ACUTE	< 0.001	0.63
			ACUTE	32.63 (17.54)			ACUTE vs POST	0.002	0.35
			POST	38.73 (17.70)			PRE vs POST	0.156	0.51
		**CG**	PRE	30.08 (10.94)	Paired t-test	0.50	-	-	-
			POST	31.07 (8.55)			-	-	-
		**Interaction**	Session*Group	-	Mixed RM-ANOVA	0.003	-	-	-
	**Duration (s)**	**WG**	PRE	26.75 (6.82)	RM-ANOVA	0.045	PRE vs ACUTE	0.041	0.33
			ACUTE	24.81 (5.56)			PRE vs POST	0.458	0.13
			POST	24.69 (4.18)			ACUTE vs POST	0.029	0.46
		**CG**	PRE	27.22 (4.95)	Paired t-test	0.09	-	-	-
			POST	25.40 (4.99)			-	-	-
		**Interaction**	Session*Group	-	Mixed RM-ANOVA	0.883	-	-	-
**Lateral Bending**	**SIL ROM (°)**	**WG**	PRE	37.60 (10.88)	RM-ANOVA	0.016	PRE vs ACUTE	0.383	0.10
			ACUTE	38.73 (10.67)			ACUTE vs POST	0.035	0.29

			POST	41.93 (11.26)			PRE vs POST	0.005	0.39
		**CG**	PRE	37.28 (7.59)	Paired t-test	0.78	-	-	-
			POST	36.99 (7.99)			-	-	-
		**Interaction**	Session*Group	-	Mixed RM-ANOVA	0.014	-	-	-
	**SIA ROM (°)**	**WG**	PRE	91.50 (12.06)	RM-ANOVA	0.880	PRE vs ACUTE	-	-
			ACUTE	90.70 (8.85)			PRE vs POST	-	-
			POST	91.56 (6.57)			ACUTE vs POST	-	-
		**CG**	PRE	89.31 (9.33)	Paired t-test	0.31	-	-	-
			POST	87.53 (6.11)			-	-	-
		**Interaction**	Session*Group	-	Mixed RM-ANOVA	0.497	-	-	-
	**Duration (s)**	**WG**	PRE	30.67 (7.05)	RM-ANOVA	< 0.001	PRE vs ACUTE	< 0.001	0.91
			ACUTE	25.25 (5.38)			ACUTE vs POST	0.076	0.37
			POST	27.47 (4.81)			PRE vs POST	0.025	0.54
		**CG**	PRE	34.66 (7.10)	Paired t-test	0.01	-	-	-
			POST	30.83 (5.13)			-	-	-
		**Interaction**	Session*Group	-	Mixed RM-ANOVA	0.039	-	-	-
**Twist**	**SRL ROM (°)**	**WG**	PRE	27.03 (10.32)	RM-ANOVA	0.018	PRE vs ACUTE	0.041	0.38
			ACUTE	31.30 (11.29)			ACUTE vs POST	0.762	0.05
			POST	31.85 (11.74)			PRE vs POST	0.029	0.42
		**CG**	PRE	25.17 (9.09)	Paired t-test	0.52	-	-	-
			POST	25.79 (8.81)			-	-	-
		**Interaction**	Session*Group	-	Mixed RM-ANOVA	0.048	-	-	-
	**SRA ROM (°)**	**WG**	PRE	78.13 (20.96)	RM-ANOVA	0.104	PRE vs ACUTE	-	-

			ACUTE	83.21 (26.66)			PRE vs POST	-	-
			POST	84.83 (22.25)			ACUTE vs POST	-	-
		**CG**	PRE	78.70 (14.33)	Paired t-test	0.13	-	-	-
			POST	82.19 (14.94)			-	-	-
		**Interaction**	Session*Group	-	Mixed RM-ANOVA	0.434	-	-	-
	**Duration (s)**	**WG**	PRE	31.83 (8.28)	RM-ANOVA	0.018	PRE vs ACUTE	0.008	0.59
			ACUTE	27.91 (5.93)			ACUTE vs POST	0.442	0.14
			POST	28.86 (4.68)			PRE vs POST	0.040	0.45
		**CG**	PRE	34.51 (8.10)	Paired t-test	0.09	-	-	-
			POST	32.35 (5.59)			-	-	-
		**Interaction**	Session*Group	-	Mixed RM-ANOVA	0.014	-	-	-
**Anterior Flexion**	**S1-L1 ROM (°)**	**WG**	PRE	69.70 (16.48)	RM-ANOVA	0.192	PRE vs ACUTE	-	-
			ACUTE	70.66 (17.31)			ACUTE vs POST	-	-
			POST	66.45 (17.27)			PRE vs POST	-	-
		**CG**	PRE	63.00 (17.36)	Paired t-test	0.74	-	-	-
			POST	63.66 (14.62)			-	-	-
		**Interaction**	Session*Group	-	Mixed RM-ANOVA	0.266	-	-	-
	**S1-L3 ROM (°)**	**WG**	PRE	56.32 (15.87)	RM-ANOVA	0.408	PRE vs ACUTE	-	-
			ACUTE	54.94 (16.31)			PRE vs POST	-	-
			POST	50.85 (15.84)			ACUTE vs POST	-	-
		**CG**	PRE	50.55 (15.84)	Paired t-test	1.00	-	-	-
			POST	50.57 (14.28)			-	-	-

		**Interaction**	Session*Group	-	Mixed RM-ANOVA	0.281	-	-	-
	**L1-T1 ROM (°)**	**WG**	PRE	99.18 (21.82)	RM-ANOVA	0.231	PRE vs ACUTE	-	-
			ACUTE	98.22 (19.04)			ACUTE vs POST	-	-
			POST	102.57 (18.32)			PRE vs POST	-	-
		**CG**	PRE	98.51 (16.96)	Paired t-test	0.85	-	-	-
			POST	98.16 (15.13)			-	-	-
		**Interaction**	Session*Group	-	Mixed RM-ANOVA	0.320	-	-	-
	**L1-T6 ROM (°)**	**WG**	PRE	93.01 (18.75)	RM-ANOVA	0.095	PRE vs ACUTE	-	-
			ACUTE	93.20 (19.77)			ACUTE vs POST	-	-
			POST	96.95 (18.59)			PRE vs POST	-	-
		**CG**	PRE	93.35 (18.24)	Paired t-test	0.74	-	-	-
			POST	93.89 (16.27)			-	-	-
		**Interaction**	Session*Group	-	Mixed RM-ANOVA	0.265	-	-	-
	**T6-T1 ROM (°)**	**WG**	PRE	101.77 (22.78)	RM-ANOVA	0.397	PRE vs ACUTE	-	-
			ACUTE	99.96 (19.63)			PRE vs POST	-	-
			POST	103.58 (17.38)			ACUTE vs POST	-	-
		**CG**	PRE	101.30 (13.96)	Paired t-test	0.71	-	-	-
			POST	100.54 (14.74)			-	-	-
		**Interaction**	Session*Group	-	Mixed RM-ANOVA	0.548	-	-	-
	**Pelvis ROM (°)**	**WG**	PRE	31.33 (19.28)	RM-ANOVA	0.165	PRE vs ACUTE	-	-
			ACUTE	29.51 (16.00)			ACUTE vs POST	-	-
			POST	28.47 (15.73)			PRE vs POST	-	-
		**CG**	PRE	21.42 (12.79)	Paired t-test	0.14	-	-	-

			POST	25.54 (13.53)			-	-	-
		**Interaction**	Session*Group	-	Mixed RM-ANOVA	0.113	-	-	-
	**Trunk ROM (°)**	**WG**	PRE	88.52 (17.53)	RM-ANOVA	0.357	PRE vs ACUTE	-	-
			ACUTE	88.25 (17.53)			ACUTE vs POST	-	-
			POST	90.84 (16.16)			PRE vs POST	-	-
		**CG**	PRE	86.71 (15.77)	Paired t-test	0.66	-	-	-
			POST	87.32 (15.23)			-	-	-
		**Interaction**	Session*Group	-	Mixed RM-ANOVA	0.547	-	-	-
	**Thorax ROM (°)**	**WG**	PRE	32.31 (12.00)	RM-ANOVA	0.365	PRE vs ACUTE	-	-
			ACUTE	29.17 (10.72)			PRE vs POST	-	-
			POST	37.17 (15.18)			ACUTE vs POST	-	-
		**CG**	PRE	38.00 (11.80)	Paired t-test	0.17	-	-	-
			POST	35.51 (11.99)			-	-	-
		**Interaction**	Session*Group	-	Mixed RM-ANOVA	0.147	-	-	-
	**Duration (s)**	**WG**	PRE	22.26 (6.57)	RM-ANOVA	0.003	PRE vs ACUTE	0.01	0.47
			ACUTE	19.72 (5.07)			ACUTE vs POST	0.624	0.08
			POST	19.30 (3.82)			PRE vs POST	0.005	0.55
		**CG**	PRE	25.54 (5.69)	Paired t-test	0.01	-	-	-
			POST	22.77 (3.80)			-	-	-
		**Interaction**	Session*Group	-	Mixed RM-ANOVA	0.029	-	-	-
